# Genetic characterization, species differentiation and detection of *Fasciola *spp. by molecular approaches

**DOI:** 10.1186/1756-3305-4-101

**Published:** 2011-06-10

**Authors:** Lin Ai, Mu-Xin Chen, Samer Alasaad, Hany M Elsheikha, Juan Li, Hai-Long Li, Rui-Qing Lin, Feng-Cai Zou, Xing-Quan Zhu, Jia-Xu Chen

**Affiliations:** 1 State Key Laboratory of Veterinary Etiological Biology, Key Laboratory of Veterinary Parasitology of Gansu Province, Lanzhou Veterinary Research Institute, CAAS, Lanzhou, Gansu Province 730046, P R China; 2 National Institute of Parasitic Diseases, Chinese Center for Disease Control and Prevention, Shanghai 200025, P R China; 3 College of Veterinary Medicine, South China Agricultural University, Guangzhou, Guangdong Province 510642, P R China; 4 Estación Biológica de Doñana, Consejo Superior de Investigaciones Científicas (CSIC), Avda. Américo Vespucio s/n 41092 Sevilla, Spain; 5 School of Veterinary Medicine and Science, University of Nottingham, Sutton Bonington Campus, Loughborough, LE12 5RD, UK; 6 College of Animal Science and Technology, Yunnan Agricultural University, Kunming, Yunnan Province 650201, P R China; 7 College of Animal Science and Veterinary Medicine, Heilongjiang Bayi Agricultural University, Daqing, Heilongjiang Province 163319, P R China

## Abstract

Liver flukes belonging to the genus *Fasciola *are among the causes of foodborne diseases of parasitic etiology. These parasites cause significant public health problems and substantial economic losses to the livestock industry. Therefore, it is important to definitively characterize the *Fasciola *species. Current phenotypic techniques fail to reflect the full extent of the diversity of *Fasciola *spp. In this respect, the use of molecular techniques to identify and differentiate *Fasciola *spp. offer considerable advantages. The advent of a variety of molecular genetic techniques also provides a powerful method to elucidate many aspects of *Fasciola *biology, epidemiology, and genetics. However, the discriminatory power of these molecular methods varies, as does the speed and ease of performance and cost. There is a need for the development of new methods to identify the mechanisms underpinning the origin and maintenance of genetic variation within and among *Fasciola *populations. The increasing application of the current and new methods will yield a much improved understanding of *Fasciola *epidemiology and evolution as well as more effective means of parasite control. Herein, we provide an overview of the molecular techniques that are being used for the genetic characterization, detection and genotyping of *Fasciola *spp..

## Background

Fascioliasis is an important food-and water-borne parasitic zoonosis caused by liver flukes of the genus *Fasciola *(Platyhelminthes: Digenea: Fasciolidae) [1,2]. *Fasciola *spp. have a cosmopolitan distribution, with high frequency in tropical areas [1,3,4]. Human fascioliasis has been reported in numerous countries [1,3,5]. It is estimated that millions of people are infected worldwide and the number of people at risk exceeds 180 million [6]. Also, fascioliasis is one of the most important parasitic diseases in grazing animals with over 700 million production animals being at risk of infection and economic losses were estimated at > US$ 2 billion per year worldwide [6].

A few species have been described within the genus *Fasciola*, but only three species, *Fasciola hepatica, Fasciola gigantica *and *Fasciola jacksoni *are commonly recognized as taxonomically valid, with *F. hepatica *mainly occurring in temperate areas, *F. gigantica *in tropical zones, and both taxa overlapping in subtropical areas [6-11]. *F. jacksoni *is known as the fasciolid of Asian elephants and its phylogenetic position is still uncertain [12]. Given the adverse impact of *Fasciola *infection on human health and its economic significance, rapid and accurate identification of *Fasciola *species is necessary for successful clinical management of infection, and for epidemiological surveys.

For a long time, the identification of *Fasciola *spp. has been based solely on traditional morphological approaches. However, due to the limitations of morphological methods, various molecular approaches have been developed and used for the identification and differentiation of *Fasciola *species. Importantly, these molecular methods have raised questions and spurred debate on the recognition of the "intermediate *Fasciola*" as a hybrid/introgressed form between *F. hepatica *and *F. gigantica *[8,13-20]. This hybrid *Fasciola *represents the emergence of a natural diversity previously undetected using conventional approaches, probably because of the inadequacy of their discriminatory power.

This article reviews molecular techniques used to identify and detect genetic variation among *Fasciola *spp.

### The identification and recognition of the "intermediate *Fasciola*"

A number of genetic and phylogenetic studies (Table 1) using different molecular targets have shown the existence of novel "intermediate *Fasciola*" [13-19,21-23]. Ribosomal DNA (rDNA) is one of the most useful markers in genetic studies because it is available in high copy number and contains variable regions flanked by more conserved regions [24]. Previous studies have demonstrated that the first and second internal transcribed spacers (ITS-1 and ITS-2) of rDNA located between the nuclear small and large subunit rRNA genes can provide genetic markers for species-level identification of *Fasciola *[14,18,21,22,25] (Table 1). The ITS-2 sequence motifs were considered the DNA barcodes for *Fasciola *spp. [26]. Comparing ITS-2 sequences, six sites at which *F. gigantica *and *F. hepatica *differ were found, and one of these is a deletion in *F. gigantica *relative to *F. hepatica *[18,22,25,27]. Whereas, the "intermediate *Fasciola*" has nucleotides shared between the two *Fasciola *species. In agreement with results obtained by using ITS-2 sequences, *F. gigantica *was found to be different from *F. hepatica *at five nucleotide positions in the ITS-1 sequences, whereas the "intermediate *Fasciola*" has the ITS-1 sequence of both *F. gigantica *and *F. hepatica *[15-17,21]. In addition to the ITS sequences, the D2 region of 28S rDNA provided genetic evidence for the existence of natural hybridization between *F. gigantica *and *F. hepatica *in Korea [18].

**Table 1:** Summary of molecular approaches used for the detection and/or genetic differentiation of *Fasciola *spp..

Molecular approach	Species investigated			Developmental stage			DNA target regions	References
	*F. hepatica*	*F. gigantica*	Intermediate form	Adult	Cercaria	Eggs		
Conventional PCR	√			√			ITS2	38
	√	√	√	√			ITS2	14, 27
	√	√		√			ITS1, ITS2	25
	√			√			*nad*1, *cox*1	28
	√	√	√	√			*nad*1, *cox*1	13, 17
	√	√	√	√			ITS2, *cox*1	9
	√	√	√	√			ITS1, *nad*1	16
	√	√	√	√			*cox*1, ITS1, ITS2	15
	√	√	√	√			*nad*1, *cox*1, ITS2, 28S	18
	√			√			Complete mitochondrial genome	30
		√			√		124 bp repetitive DNA sequence	49
	√				√		124 bp repetitive DNA sequence	50
	√			√			28S	39
Multiplex PCR	√				√		*cox*1, ITS1, ITS2	52
Specific PCR	√	√	√	√	√	√	ITS2	47
PCR-RFLP	√	√	√	√			ITS2	22
		√		√			*cox*1, ITS2	23
	√	√	√	√			ITS1	8, 21
PCR-SSCP	√	√		√			Repetitive DNA sequences	46
RAPD-PCR		√		√			Random nucleotide sequence	43
	√	√	√	√			Random nucleotide sequence	53
SRAP	√			√			Random nucleotide sequence	42
	√	√	√	√			Random nucleotide sequence	10
DNA probe		√			√		Repetitive DNA fragments	48
TaqMan real-time PCR	√	√	√	√			ITS2	54
LAMP	√	√	√	√	√	√	IGS	58
PCR	√			√			Microsatellites	59

Other molecular markers being used frequently are mitochondrial DNA (mtDNA) sequences. Almost all of eukaryotes contain a mitochondrial genome which evolves at a faster rate than the nuclear genome and, is thus suitable for discriminating closely related organisms [28-30], especially at the species and sub-species levels [31-33]. mtDNA sequence analysis also provided evidence for the existence of the "intermediate *Fasciola*" [15,16,19].

### Genetic variation among and within *Fasciola *spp

Revealing genetic variation has been the focus of many studies because accurate analysis of genetic variability has important implications for studying population biology, epidemiology, and genetic structure of these parasites, and thus the efficient control of the diseases they cause.

Examination of *Fasciola *specimens from geographical areas where *F. hepatica *and *F. gigantica *co-exist, such as Egypt and Iran, demonstrated the existence of phenotypic variations in adult flukes [20,34]. Molecular approaches utilizing a number of genetic makers are useful for genetic characterization and studies of genetic variability among parasite populations [35-37]. A recent study investigated the extent of genetic variation among *Fasciola *collected from different host species and geographical localities in Spain using ITS rDNA as genetic markers, and concluded that only a single species *F. hepatica *exists in Spain, although a slight sequence variation in the ITS-2 was detected among *F. hepatica *samples from different host species and geographical areas [38]. Spanish *F. hepatica *examined in that study differed from *F. hepatica *from elsewhere by two nucleotides in the ITS-2 [38]. Another study detected some genetic variations in *F. hepatica *from northwest of Spain using the 28S rDNA as genetic marker, and there were nucleotide differences in a number of sequence positions [39].

Sequence related amplified polymorphism (SRAP) is a molecular technique for detecting genetic variation in the open reading frames (ORFs) of genomes of related organisms [40,41]. A recent study showed that the SRAP technique was useful for revealing genetic variability within and between *F. hepatica, F. gigantica *and the "intermediate *Fasciola*", which substantiated the evidence for the existence of the "intermediate *Fasciola*" [10]. Using the same technique, genetic variability among a number of *F. hepatica *samples collected from six host species and 16 geographical locations in Spain was investigated [42], and a low genetic variation in the coding regions of the genomes was found, indicating the lack of genetic association between *F. hepatica *and their hosts and/or geographical locations in Spain [42].

Randomly amplified polymorphic DNA (RAPD) is a useful genetic marker for the identification and genetic characterization of parasite populations [37]. RAPD is a useful technique for the identification and differentiation of *F. hepatica *and *F. gigantica*. Using the RAPD technique, some degree of genetic variation was detected among *F. gigantica *isolates from cattle, buffalo, and goat. Cattle and buffalo isolates of *F. gigantica *showed 100% homogeneity, whereas goat and cattle/buffalo isolates displayed 92.68% similarity [43].

mtDNA sequences provided useful markers for studies of genetic variability and population structures [28,44]. Walker et al. (2007) examined DNA polymorphism in the entire mitochondrial genome and showed that genetic diversity exist among and within *F. hepatica *populations from cattle and sheep and provided evidence for the existence of multiple mitochondrial lineages within infra-populations of *F. hepatica *[44]. Another study examined genetic variation in eastern European and western Asian populations of *F. hepatica *using partial mitochondrial NADH dehydrogenase subunits 1 (*nad*1) and cytochrome *c *oxidase subunit 1 gene (*cox*1) as genetic markers, and revealed the existence of two well-defined lineages with two main haplotypes and a number of shared divergent haplotypes among the examined *F. hepatica *populations [28].

### Molecular detection and identification of *Fasciola *spp

In order to overcome the limitation of the phenotypic methods, genotypic approaches have been used for the identification and differentiation of *Fasciola *spp. [9,10,21,22,45-47]. Before the availability of PCR-based approaches, DNA probes were the alternative choice for the genotypic detection of *Fasciola *spp. [48]. However, DNA probe-based assays usually require the use of radioactive isotopes and can have bio-safety concerns.

Over the last two decades, several PCR-based approaches (Table 1), including PCR-linked restriction fragment length polymorphism (PCR-RFLP), PCR-linked single-strand conformation polymorphism (PCR-SSCP) and specific PCR assays, have been developed for the accurate identification of *Fasciola *spp. [8,21,22,45,46]. For example, a simple and rapid PCR-RFLP assay targeting a 618-bp sequence of the 28S rDNA was developed for the differentiation between *F. hepatica *and *F. gigantica *[45]. A similar PCR-RFLP assay using restriction endonucleases *Hsp*92 II and *Rca *I was developed to differentiate between *F. hepatica, F. gigantica *and the "intermediate *Fasciola*" in China utilizing the ITS-2 rDNA as genetic marker [22]. Using the ITS-1 rDNA as genetic marker, Lin et al. (2007) established a PCR-SSCP assay for the accurate identification and differentiation between *F. hepatica, F. gigantica *and the "intermediate *Fasciola*" [21]. A very recent study established a fluorescence-based SSCP (F-PCR-SSCP) for the identification of *Fasciola *spp. [46].

Recently, several specific PCR assays have been developed to differentiate *F. hepatica *from *F. gigantica *and detect *Fasciola *infections in the intermediate host snail and definitive hosts (such as buffalo), utilizing various genetic markers, such as *cox*1, ITS, non-coding repetitive DNA fragment as well as RAPD-derived sequences [47,49-53]. For example, Ai et al. (2010) established a specific PCR method based on the ITS-2 sequences to identify *F. hepatica, F. gigantica *and the "intermediate *Fasciola*". The method was sensitive as it was able to amplify target DNA fragment from a single *Fasciola *egg [47]. Specific PCR assays, using two primer sets derived from RAPD-derived sequences from English *F. hepatica *and Ghanaian *F. gigantica*, were able to distinguish *F. hepatica *from *F. gigantica *from cattle and sheep hosts from different countries [53]. Alasaad et al. (2011) developed a highly specific, sensitive, and simple TaqMan-based real-time PCR assay for the identification of *F. hepatica *and *F. gigantica*, as well as the "intermediate *Fasciola*" based on sequences of the ITS-2 rDNA [54].

A PCR assay was used to detect *F. gigantica *infection in the snail vector host, *Lymnaea auricularia *[49]. The specific primers amplified a *F. gigantica *specific 124-bp non-coding repetitive DNA fragment from infected *L. auricularia *snails. Kaplan et al. (1995) also identified a 124-bp repetitive DNA sequence which was used as a specific probe for detection of *F. hepatica *infections in intermediate host snails (*Fossaria cubensis *and *Pseudosuccinea columella*) [51]. A multiplex PCR assay was able to detect *F. hepatica *DNA in *L. viatrix *snails, which were even formalin-fixed and paraffin-embedded [52]. TaqMan chemistry was adopted by Schweizer et al. (2007) to establish a real-time PCR assay. The combined use of primers and probe targeting an 86-bp target of a repetitive 449-bp genomic DNA fragment facilitated the detection of *L. truncatula *naturally infected with *F. hepatica*. These PCR assays are highly specific and sensitive, providing useful and practical tools for the epidemiological investigation of *Fasciola *in the snail hosts [55].

Loop-mediated isothermal amplification (LAMP) allows amplification of target nucleic acids under isothermal conditions with high sensitivity, specificity, rapidity and precision, which has found broad applications for the detection of pathogens [56,57]. Ai et al. (2010) developed a LAMP assay for the sensitive and rapid detection and discrimination of *F. hepatica *and *F. gigantica*. The assay can be done in 45 min under isothermal conditions at 61°C or 62°C by employing a set of 4 species-specific primer mixtures and the results can be checked visually. This LAMP assay was approximately 10^5 ^times more sensitive than the conventional specific PCR assays, and may find applicability in the field settings or in poorly-equipped laboratories in endemic countries [58].

### Conclusions and future perspectives

Much of the current knowledge of *Fasciola *spp. taxonomy and epidemiology has stemmed from numerous observational and morphological studies. However, conventional methods of detection and differentiation of *Fasciola *do not accurately reflect the full diversity of *Fasciola *spp. Nevertheless, molecular genetics studies over the past two decades have added significantly to our understanding of *Fasciola *taxonomy, genetics, and contributed to the development of advanced approaches for the accurate identification and differentiation of *Fasciola *spp. Importantly, these molecular methods have facilitated the identification of the hybrid "intermediate *Fasciola*". However, presently there is no molecular diagnostic method (e.g. a Copro-PCR) developed and validated for use with human stools.

We are still far from a complete understanding of the molecular evolution of the hybrid *Fasciola*, and many questions remain unanswered. For example, what is the outcome of experimental crosses between *F. hepatica *and *F. gigantica*? What are the differences between the "intermediate *Fasciola*" and other *Fasciola *species at the genomic and transcriptomic levels? Comprehensive genetic characterization using more variable markers such as microsatellites (eg. [59]) along with transcriptional analysis of *Fasciola *species can be used to refine the taxonomic status of the "intermediate *Fasciola*" and to assess its potential as a zoonotic agent.

On the analytical methods front, there is a clear need for the application of high-throughput molecular techniques such as next-generation sequencing, transcriptomics, proteomics and large-scale analysis of single nucleotide polymorphisms. The successful application of these and other techniques should bring more insights into the population genetic structure and the evolutionary process in *Fasciola*.

Recently, there have been increasing interests in the studies of transcriptome and proteome of *F. hepatica *[60-62]. More studies in these promising areas of research will expand our understanding of the complex biology of different *Fasciola *species, which, in turn, will facilitate the development of novel means of therapeutic and immunological intervention.

## Competing interests

The authors declare that they have no competing interests.

## Authors' contributions

XQZ, JXC, SA and HME conceived and designed the review, and critically revised the manuscript. LA and MXC drafted the manuscript. JL, HLL, RQL and FCZ contributed to drafting the manuscript. All authors read and approved the final manuscript.
